# Assessment of hyperparathyroidism secondary to CKD beyond laboratory tests: the important validation of the Parathyroid Assessment of Symptom (PAS) questionnaire for Portuguese language (Brazil)

**DOI:** 10.1590/2175-8239-JBN-2024-E009en

**Published:** 2024-11-25

**Authors:** Fellype Carvalho Barreto, Cássia Gomes da Silveira Santos

**Affiliations:** 1 Universidade Federal do Paraná, Departamento de Clínica Médica, Disciplina de Nefrologia, Curitiba, PR, Brazil.; 2 Universidade Federal do Paraná, Empresa Brasileira de Serviços Hospitalares (EBSERH), Complexo Hospital de Clínicas, Serviço de Nefrologia, Curitiba, PR, Brazil.; 3 Universidade Federal do Paraná, Departamento de Clínica Médica, Programa de Pós-Graduação em Medicina Interna e Ciências da Saúde, Curitiba, PR, Brazil.

Secondary hyperparathyroidism (SHPT) is a frequent complication that may be present from the early stages of chronic kidney disease (CKD)^1^. It is estimated that the prevalence of SHPT, depending on diagnostic criteria and CKD stage, ranges from 31% to 85%^2^, reinforcing the extent of its impact on chronic kidney disease patients, and the importance of appropriate management. SHPT is primarily characterized by elevated parathyroid hormone levels, often accompanied by hypocalcemia and hyperphosphatemia; it is associated with dysfunction in several organs and systems, such as the cardiovascular, musculoskeletal, hematopoietic, and nervous systems, among others. Consequently, patients with SHPT may present with various clinical complaints, such as pruritus, arthralgia, bone pain, myalgia, fatigue, and muscle weakness^1^. Therefore, it is understandable that, due to this myriad of alterations and manifestations, SHPT contributes to reduced survival and quality of life among individuals with CKD^1^.

Quality of life is considered an important indicator of how a disease could affect people’s lives and for assessing healthcare quality, particularly in chronic diseases. It has been increasingly used for therapeutic decision-making, health policies, and reimbursement decisions^3^. However, the difficulty in quantifying quality of life, and its perception as a “surrogate” outcome - as opposed to “hard” outcomes and laboratory tests that can be measured numerically – pose an obstacle to implementing its assessment both in practice and clinical research^3^. Additionally, it has been reported that the use of disease-specific quality of life questionnaires is preferable to generic questionnaires, so as to provide a better understanding of the impact related to a particular illness^4^.

In line with these issues, namely the importance of the impact of HPT on CKD and the need for a specific questionnaire, the study by Campos et al.^5^ aimed to validate the use of the Parathyroid Assessment of Symptom (PAS) questionnaire in Brazil to assess the quality of life of CKD patients with hyperparathyroidism. Following a thorough translation and cultural adaptation, the PAS questionnaire was administered to 100 patients (61 men; age 55.6 ± 15.1 [21–81] years), with 68% having SHPT, and 32% having THPT. The majority of patients were overweight, and in 22 patients, diabetes *mellitus* was considered the cause of CKD. Among the patients with SHPT, most were on hemodialysis or peritoneal dialysis; approximately 12% were undergoing conservative treatment. Thirty-two patients were kidney transplant recipients with tertiary HPT (THPT). For validating the PAS questionnaire, the Short form health-36 (SF-36) questionnaire was used as the gold standard. According to the authors, the reliability of the PAS questionnaire was demonstrated, in addition to the consistency and agreement of both questionnaires. The authors suggest the potential applicability of the Brazilian Portuguese version of PAS to identify symptoms and assess improvements in quality of life after pharmacological or surgical therapy for SHPT^5^.

The PAS questionnaire was developed by Professor Pasieka in 1998, to assess the impact of primary HPT (PHPT) on patients’ lifestyle and well-being. Using this tool, it was possible to demonstrate improved clinical manifestations in patients with PHPT^6^ and, subsequently, in patients with SHPT and THPT undergoing surgical treatment^7^. These studies illustrate the potential applicability of the questionnaire and underscore the importance of having this tool translated and validated for our context, as has already been done in other countries^8^.

Given the potential relevance of the study by Campos et al.^5^, it is important to consider a few points related to the study design as a stimulus for future research using the PAS questionnaire. Firstly, it is essential to keep in mind that the manifestations assessed by the PAS questionnaire are not specific to hyperparathyroidism and that this instrument was originally developed to assess PHPT. The present study included a heterogeneous group of patients regarding CKD stage, renal replacement therapy (RRT) modality, and PTH level. Within this context, we should consider that there may be an overlap between uremic symptoms in general and those related to hyperparathyroidism. Furthermore, age and comorbidities, such as overweight and diabetes *mellitus*, as well as medications, a potential confounding factor not reported by the authors, could directly interfere with patient symptomatology. Indeed, a recent study reported that mild to moderately elevated PTH levels in elderly patients with advanced CKD, not on RRT, were associated with a lower symptom burden compared to those of patients with severe hyperparathyroidism^9^. Thus, for a better appreciation of PAS consistency in assessing CKD patients, it would have been interesting to analyze, for example, groups of patients separated by PTH range and by RRT modality or CKD stage. This would help demonstrating that PAS could in fact capture SHPT quality of life and symptomatology in comparison to the SF-36. This approach could potentially provide a clearer demonstration of the contribution of HPT to the assessed symptoms and, consequently, the reliability and applicability of the PAS questionnaire as a HPT-specific quality of life questionnaire for chronic kidney disease patients.

The translation, cultural adaptation, and validation of the PAS questionnaire represent a major contribution to the complementary assessment of chronic kidney disease patients with HPT. From now on, we have the opportunity to incorporate this tool into clinical practice and further studies.
