# Supplementary material for: Assessment of hyperparathyroidism secondary to CKD beyond laboratory tests: the important validation of the Parathyroid Assessment of Symptom (PAS) questionnaire for Portuguese language (Brazil)
Source: J Bras Nefrol. 2024 Nov 25;46(4):e2024E009. doi: 10.1590/2175-8239-JBN-2024-E009en (PMC11588316; doi:10.1590/2175-8239-JBN-2024-E009en)
Supplement: Translation

# Translation: Avaliação do hiperparatireoidismo secundário à DRC além dos exames laboratoriais: a importante validação do questionário *Parathyroid Assessment of Symptom* (PAS) para a língua portuguesa

**DOI:** 10.1590/2175-8239-JBN-2024-E009pt

**Authors:** Fellype Carvalho Barreto, Cássia Gomes da Silveira Santos

**Affiliations:** 1 Universidade Federal do Paraná, Departamento de Clínica Médica, Disciplina de Nefrologia, Curitiba, PR, Brasil.; 2 Universidade Federal do Paraná, Empresa Brasileira de Serviços Hospitalares (EBSERH), Complexo Hospital de Clínicas, Serviço de Nefrologia, Curitiba, PR, Brasil.; 3 Universidade Federal do Paraná, Departamento de Clínica Médica, Programa de Pós-Graduação em Medicina Interna e Ciências da Saúde, Curitiba, PR, Brasil.

O hiperparatireoidismo secundário (HPTS) é uma complicação frequente e que pode estar presente desde as fases iniciais da doença renal crônica (DRC)^1^. Estima-se que a prevalência do HPTS, a depender do critério diagnóstico e da fase da DRC, varia de 31% a 85%^2^, o que reforça a extensão de seu impacto nos pacientes renais crônicos e a importância do manejo adequado. O HPTS é caracterizado, primordialmente, por níveis elevados do paratormônio, além de, comumente, hipocalcemia e hiperfosfatemia; está associado à disfunção de diversos órgãos e sistemas, como o cardiovascular, o musculoesquelético, o sistema hematopoiético, o sistema nervoso, entre outros. Por conseguinte, os pacientes com HPTS podem apresentar diversas queixas clínicas, como prurido, artralgia, dores ósseas, mialgia, fadiga e fraqueza muscular^1^. Portanto, é compreensível que, em razão dessa miríade de alterações e manifestações, o HPTS contribua para reduzir a sobrevida e a qualidade de vida dos indivíduos com DRC^1^.

A qualidade de vida é considerada um importante indicador de como uma doença pode afetar a vida das pessoas e de avaliação da qualidade da atenção à saúde, particularmente nas doenças crônicas, que tem sido cada vez mais utilizada para decisões terapêuticas, políticas de saúde e decisões sobre reembolso^3^. Todavia, o fato de a qualidade de vida ser de difícil mensuração quantitativa e, por vezes, percebida como um desfecho “suave”, em oposição aos desfechos “duros” e exames laboratoriais que podem ser aferidos numericamente, representa um obstáculo para a implementação da sua avaliação tanto na prática quanto na pesquisa clínica^3^. Adicionalmente, tem sido reportado que a aplicação de questionários de qualidade de vida específicos para uma determinada doença é preferível, em comparação aos questionários genéricos, para fornecer melhor compreensão do impacto relacionada a uma determinada enfermidade^4^.

Em consonância com essas questões, isto é, a importância do impacto do HPT na DRC e de se utilizar um questionário específico, o estudo de Campos et al.^5^ objetivou validar a utilização no Brasil do questionário *Parathyroid Assessment of Symptom* (PAS) para avaliar a qualidade de vida dos pacientes com DRC e hiperparatireoidismo. Após criteriosa tradução e adaptação cultural, o questionário PAS foi aplicado em 100 pacientes (61 homens; idade 55,6 ± 15,1 [21–81] anos), 68% com HPTS e 32% com HPTT. A maioria dos pacientes apresentava sobrepeso, e em 22 pacientes o diabetes *mellitus* foi considerado a causa da DRC. Entre os pacientes com HPTS, a maioria estava em hemodiálise ou em diálise peritoneal; cerca de 12% estavam em tratamento conservador. Trinta e dois pacientes eram transplantados renais com o HPT terciário (HPTT). Para a validação do questionário PAS, foi utilizado como padrão-ouro o questionário *Short form health-36* (SF-36). De acordo com os autores, foi demonstrada a confiabilidade do questionário PAS, além da consistência e da concordância de ambos os questionários. Os autores sugerem a potencial aplicabilidade da versão em português do PAS para identificar sintomas e avaliar melhorias na qualidade de vida após a terapia farmacológica ou cirúrgica para o HPTS^5^.

O questionário PAS foi desenvolvido pelo professor Pasieka, em 1998, com o objetivo de avaliar o impacto do HPT primário (HPTP) no estilo de vida e no bem-estar dos pacientes. Por meio dessa ferramenta, foi possível demonstrar a melhoria de manifestações clínicas de pacientes com HPTP^6^ e, subsequentemente, de pacientes com HPTS e HPTT submetidos a tratamento cirúrgico^7^. Esses estudos ilustram a potencial aplicabilidade do questionário e a importância de termos esse instrumento traduzido e validado para o nosso meio, conforme já foi feito em outros países^8^.

Dada a potencial relevância do estudo de Campos et al.^5^, é importante ter atenção a alguns pontos relacionados ao desenho do estudo como estímulo para futuras pesquisas que venham a utilizar o questionário PAS. Primeiramente, é preciso ter em mente que as manifestações avaliadas pelo questionário PAS não são específicas do hiperparatireoidismo e que esse instrumento foi desenvolvido originalmente para avaliar o HPTP. O presente estudo incluiu um grupo heterogêneo de pacientes com relação ao estágio da DRC, modalidade de terapia renal substitutiva (TRS) e nível de PTH. Dentro desse contexto, devemos considerar que pode haver sobreposição entre os sintomas urêmicos em geral e os relacionados ao hiperparatireoidismo. Além disso, a idade e as comorbidades, como sobrepeso e diabetes *mellitus*, além de medicações, um potencial fator de confusão não reportado pelos autores, podem interferir diretamente na sintomatologia dos pacientes. De fato, um estudo recente reportou que níveis de PTH leve a moderadamente elevados em pacientes idosos com DRC avançada, não em TRS, se associou com menor carga de sintomas quando comparados aos de pacientes com hiperparatireoidismo grave^9^. Assim, teria sido interessante, para melhor apreciação da consistência do PAS na avaliação de pacientes com DRC, analisar, por exemplo, grupos de pacientes separados por faixa de PTH e por modalidade de TRS ou estágio de DRC a fim de demonstrar que, de fato, o PAS consegue capturar a qualidade de vida e sintomatologia do HPT em comparação ao SF-6. Essa abordagem poderia, potencialmente, demonstrar com maior clareza a contribuição do HPT nos sintomas avaliados e, consequentemente, a confiabilidade e aplicabilidade do questionário PAS, como questionário de qualidade de vida específico do HPT em pacientes renais crônicos.

A tradução, a adaptação cultural e a validação do questionário PAS é uma grande contribuição para a avaliação complementar dos pacientes renais crônicos com HPT. De agora em diante, temos a oportunidade de incorporar essa ferramenta na prática clínica e em futuros estudos.
